# shinyDeepGxP: a user-friendly R shiny app for predicting surface protein abundance from scRNA-seq expression using deep learning in blood cells

**DOI:** 10.1093/bioadv/vbag203

**Published:** 2026-07-25

**Authors:** Hui-Mei Tsai, Tzu-Hung Hsiao, Yu-Ching Hsu, Li-Ju Wang, Yu-Chiao Chiu, Eric Y Chuang, Yidong Chen

**Affiliations:** Graduate Institute of Biomedical Electronics and Bioinformatics, National Taiwan University, Taipei 106319, Taiwan; Greehey Children’s Cancer Research Institute, University of Texas Health San Antonio, San Antonio, TX 78229, United States; Department of Medical Research, Taichung Veterans General Hospital, Taichung 407219, Taiwan; Department of Medical Research, Taichung Veterans General Hospital, Taichung 407219, Taiwan; Department of Public Health, Fu Jen Catholic University, New Taipei City 242062, Taiwan; Institute of Genomics and Bioinformatics, National Chung Hsing University, Taichung 402202, Taiwan; Department of Medical Research, Taichung Veterans General Hospital, Taichung 407219, Taiwan; Department of Urology, James Buchanan Brady Urological Institute, Johns Hopkins University School of Medicine, Baltimore, MD 21287, United States; UPMC Hillman Cancer Center, University of Pittsburgh, Pittsburgh, PA 15232, United States; UPMC Hillman Cancer Center, University of Pittsburgh, Pittsburgh, PA 15232, United States; Department of Medicine, School of Medicine, University of Pittsburgh, Pittsburgh, PA 15232, United States; Graduate Institute of Biomedical Electronics and Bioinformatics, National Taiwan University, Taipei 106319, Taiwan; Biomedical Technology and Device Research Laboratories, Industrial Technology Research Institute, Hsinchu County 310401, Taiwan; Research and Development Center for Medical Devices, National Taiwan University, Taipei 106319, Taiwan; Department of Electrical Engineering, College of Electrical Engineering and Computer Science, National Taiwan University, Taipei 106319, Taiwan; Greehey Children’s Cancer Research Institute, University of Texas Health San Antonio, San Antonio, TX 78229, United States; Department of Population Health Sciences, University of Texas Health San Antonio, San Antonio, TX 78229, United States

## Abstract

**Motivation:**

Understanding accurate immune cell heterogeneity and function in single-cell datasets requires access to protein-level information, which is often unavailable due to experimental limitations.

**Results:**

We present shinyDeepGxP, an interactive web application featuring our deep learning model, DeepGxP, for predicting surface protein abundance from single-cell RNA-sequencing (scRNA-seq) data. This platform makes DeepGxP accessible to researchers without programming skills. Users can upload scRNA-seq count matrices and use “Predict Protein” to predict the abundance of 224 biologically relevant surface proteins. shinyDeepGxP provides visualizations to help identify distinct cell populations based on predicted protein profiles. Moreover, users can choose “Explore Model” to reveal key RNA predictors and their associated biological pathways for each protein. Overall, shinyDeepGxP is a user-friendly, freely available web tool that provides protein-level detail for RNA-only single-cell datasets, enabling multimodal discovery without additional experiments.

**Availability and implementation:**

shinyDeepGxP can be launched on https://shiny.crc.pitt.edu/deepgxp/.

## 1 Introduction

Single-cell RNA-sequencing (scRNA-seq) has transformed our understanding of cellular heterogeneity by enabling transcriptional profiling at the single-cell level (Van de Sande *et al*. 2023, Parween *et al*. 2025). Despite generating information-rich profiles, transcriptomic data alone cannot fully characterize cellular identity and function. Cellular phenotypes, such as activation status, functional state, and lineage commitment, are mediated at the protein level. Moreover, RNA expression and protein abundance often do not correlate well, making it inappropriate to infer protein abundance directly from RNA expression (Liu *et al*. 2016).

Cellular Indexing of Transcriptomes and Epitopes by sequencing (CITE-seq) overcomes these limitations by simultaneously quantifying RNA expression and protein abundance within the same cell using antibody-derived tags (ADTs) (Stoeckius *et al*. 2017). This multi-omic approach improves cell-type resolution, especially in immunology, where protein markers often serve as the gold standard for cell identification (Stoeckius *et al*. 2017, Hao *et al*. 2021), and has enabled the detection of rare or transitional cell populations in autoimmune, oncology, and infectious diseases (Kotliarov *et al*. 2020, Sparks *et al*. 2023, Parween *et al*. 2025). For instance, CD4+ and CD8+ cell clusters that overlap transcriptionally can be distinctly identified by their protein data but not by RNA data (Hao *et al*. 2021). Nevertheless, CITE-seq remains constrained by technical complexity and high cost, making it inaccessible for many laboratories and large-scale studies. As a result, most single-cell datasets include only RNA data and lack matched protein data.

To gain protein-level information from scRNA-seq, we previously developed DeepGxP, a deep learning model trained on peripheral blood mononuclear cell (PBMC) CITE-seq datasets to predict the abundance of 224 surface protein markers from whole-transcriptome profiles (Tsai *et al*. 2026). As DeepGxP was trained on PBMC CITE-seq data, it is optimized for immune cell populations, and its application to non-blood tissues or biologically distinct cell types may result in reduced prediction accuracy and should be interpreted with caution. Its predictive performance and benchmarking have been evaluated in our prior study (Tsai *et al*. 2026). However, like other protein prediction frameworks, DeepGxP is implemented via Python-based pipelines that require command-line execution, which may limit its accessibility to researchers without computational expertise. Several Shiny-based tools, including CITEViz (Kong *et al*. 2024) and CiteFuse (Kim *et al*. 2020), provide interactive web interfaces for multimodal CITE-seq analysis and visualization. However, because they depend on paired RNA and protein measurements, they are not suitable for transcriptome-only datasets.

Here, we present shinyDeepGxP, an interactive Shiny web application that deploys the previously developed and validated DeepGxP framework as an accessible, end-to-end analytical platform. Unlike the original DeepGxP study, which focused on model development, training, and benchmarking, shinyDeepGxP focuses on software implementation and accessibility. It enables users to predict 224 surface proteins directly from scRNA-seq data using a validated model without requiring paired protein measurements, software installation, or command-line usage. Beyond protein prediction, shinyDeepGxP displays model-derived interpretation results generated by DeepEnrich, an integrated gradient-based framework that identifies transcriptomic predictors and their enrichment in associated biological pathways and functions for each protein prediction. The platform also provides automated preprocessing, visualization, and downstream analysis within a unified interface. By lowering the technical barrier to applying a complex multi-omic predictive framework, shinyDeepGxP expands the practical utility of scRNA-seq data and provides a reproducible, user-friendly environment for protein-informed single-cell analysis without additional experimental costs.

## 2 Methods

### 2.1 Overview

shinyDeepGxP was developed to make the DeepGxP model accessible through a web-based interface, enabling RNA-to-protein prediction and downstream analysis of single-cell profiles without requiring programming or deep learning expertise. The application contains two modules: **Module 1—Explore Model** and **Module 2—Predict Proteins**. These two modules provide transparent exploration of model behavior while enabling protein prediction on new datasets.

Module 1 allows users to explore how DeepGxP generates protein predictions by accessing precomputed model interpretation results from the original training dataset. This module does not accept user-uploaded data.

Module 2 allows users to upload their own single-cell RNA expression data for protein prediction and multimodal analysis, with automated preprocessing.

**Figure 1:**
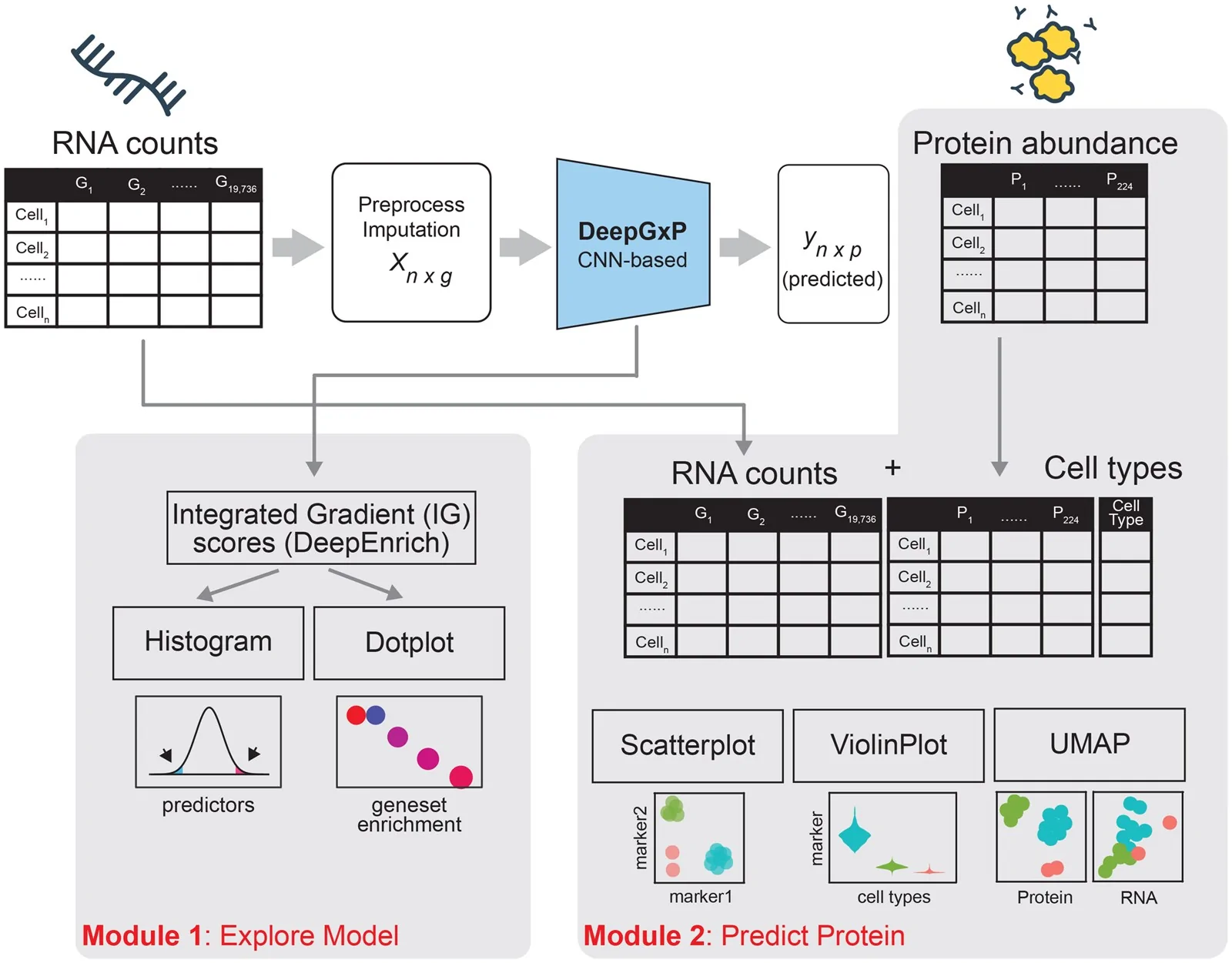
Overview of the shinyDeepGxP web application. A schematic illustration of the shinyDeepGxP workflow for predicting and interpreting surface protein abundance from single-cell RNA expression data. The input RNA expression matrix (X_*n*×__*g*_), where *n* denotes the number of cells and *g* denotes the number of genes, undergoes preprocessing and imputation before being used by DeepGxP to predict the protein abundance matrix (*Y*_*n*×*p*_), where *p* denotes the number of surface proteins. The web application contains two interactive modules. *Module 1 (Explore Model)* provides integrated gradient-based feature attribution and gene set enrichment analysis to show transcriptomic predictors for each protein prediction. *Module 2 (Predict Protein)* enables users to upload scRNA-seq data and visualize predicted protein abundances using scatterplots, violin plots, and UMAP projections, allowing comparisons across cell types and between RNA and protein levels. Together, these modules enable both interpretation of the DeepGxP model and application of protein prediction to new single-cell datasets.

An overview of shinyDeepGxP is shown in Fig. 1.

### 2.2 DeepGxP model

The core predictive engine of shinyDeepGxP is the single-cell version of DeepGxP, a convolutional neural network (CNN)-based framework developed to model RNA-protein relationships (Tsai *et al*. 2026). DeepGxP employs a one-dimensional CNN architecture consisting of a Conv1D layer, max-pooling, a flattening layer, a fully connected dense layer, and a multi-output regression layer to predict surface protein abundance. The model takes 19 738 genes as input and predicts 224 surface proteins simultaneously.

DeepGxP was trained on PBMC CITE-seq data (GSE164378) using 53 364 cells. The training dataset consisted of PBMCs and included major immune cell populations, including B cells, CD4 T cells, CD8 T cells, NK cells, monocytes, dendritic cells, and other lymphocyte subsets. The training data were neither cancer-specific nor tumor cell-specific. RNA count data were normalized with SCTransform, followed by MAGIC imputation to mitigate sparsity and dropout effects. Protein abundance was normalized using a centered log-ratio (CLR) transformation. The model was trained in a supervised manner to minimize mean squared error (MSE) between predicted and measured protein abundance, with 80–10–10 data splits and cross-validation to assess performance and prevent overfitting.

Within shinyDeepGxP, the trained model weights are fixed, and no retraining is performed during user sessions. Detailed descriptions of model architecture, preprocessing, hyperparameter optimization and benchmarking are provided in our previous DeepGxP study (Tsai *et al*. 2026).

### 2.3 DeepEnrich interpretation method

Gene-level contributions to each protein prediction were quantified using integrated gradients (IG). To focus on the most biologically relevant signals, IG values were averaged across the top 10% of samples with the highest measured protein abundance for each protein. The averaged IG values were then z-transformed to obtain normalized integrated gradient (nIG) scores across all genes.

Genes were selected for downstream pathway enrichment analysis based on a statistical threshold, with those having nIG values exceeding ±1.96 (corresponding to *P* < .05 under a normality assumption) defined as significant contributors. Positively and negatively contributing genes were categorized as positive and negative leading-edge (pLEG and nLEG) predictors, respectively, and used as input for over-representation analysis across multiple gene set databases.

A more detailed description of the DeepEnrich methodology, including the IG formulation and normalization procedures, is presented in our original study (Tsai *et al*. 2026).

### 2.4 Module 1—explore model

Module 1 enables exploration of gene-level contributions underlying DeepGxP predictions using DeepEnrich, an integrated gradient (IG)-based interpretation framework developed as part of DeepGxP (Tsai *et al*. 2026). IG scores quantify the contribution of each input gene (*n* = 19 738) to the predicted abundance of each surface protein (*n* = 224) in the training dataset (53 364 PBMC cells). Scores were precomputed and summarized across eight PBMC cell types annotated in the original study (Hao *et al*. 2021).

To allow comparison across genes, IG scores were normalized within each cell type using a z-transformation, generating normalized integrated gradient (nIG) scores that rank gene-level contribution to each protein prediction. Genes with high nIG scores were further analysed for pathway enrichment using Gene Ontology (GO), KEGG, Reactome, Hallmark, and MSigDB gene sets. Within the interface, users can select cell types, proteins, and gene set databases to explore ranked predictors and enriched pathways displayed as tables and dot plots.

### 2.5 Module 2—predict proteins

Module 2 is the primary query interface in shinyDeepGxP. Users upload gene expression count data in h5Seurat format with official gene symbols. Uploaded datasets undergo automated preprocessing consistent with the DeepGxP training pipeline, including SCTransform normalization and MAGIC imputation to mitigate RNA dropout effects.

Gene symbols are mapped to the 19 738 genes used during model training, and missing genes are set to zero after imputation to maintain compatibility with the fixed model feature space. Alternative gene aliases are resolved using ClusterProfiler’s bitr() function.

The pre-trained DeepGxP model then predicts the abundance of 224 surface proteins for each cell. RNA and predicted protein data are then analysed both independently and jointly. For multimodal integration, RNA and predicted protein matrices are combined using Seurat’s weighted-nearest neighbor (WNN) framework. Clustering is performed using the Louvain algorithm, and UMAP embeddings are generated for visualization.

Cell type annotation is performed using reference-based annotations implemented in Seurat’s TransferData() function, which assigns cell identities based on the same PBMC reference dataset used during model training.shinyDeepGxP is designed for human PBMC datasets and is consistent with the training data used to develop DeepGxP. Application to other cell types should be interpreted with caution due to potential domain differences.

### 2.6 R And web environment of DeepGxP

shinyDeepGxP was implemented in R (v4.4.2) using the Shiny framework (v1.10.0). Deep learning computations were performed using Keras (v2.15.0) and a TensorFlow backend (v2.12.0) running on Python (v3.11.5) via reticulate. Single-cell processing and visualization were conducted using Seurat (v5.2.1). RNA imputation was implemented using Rmagic (v2.0.3) which interfaces with magic-impute (v3.0.0). The application is deployed on a virtual machine with 8 CPU cores and 48 GB RAM.

## 3 Results

**Figure 2:**
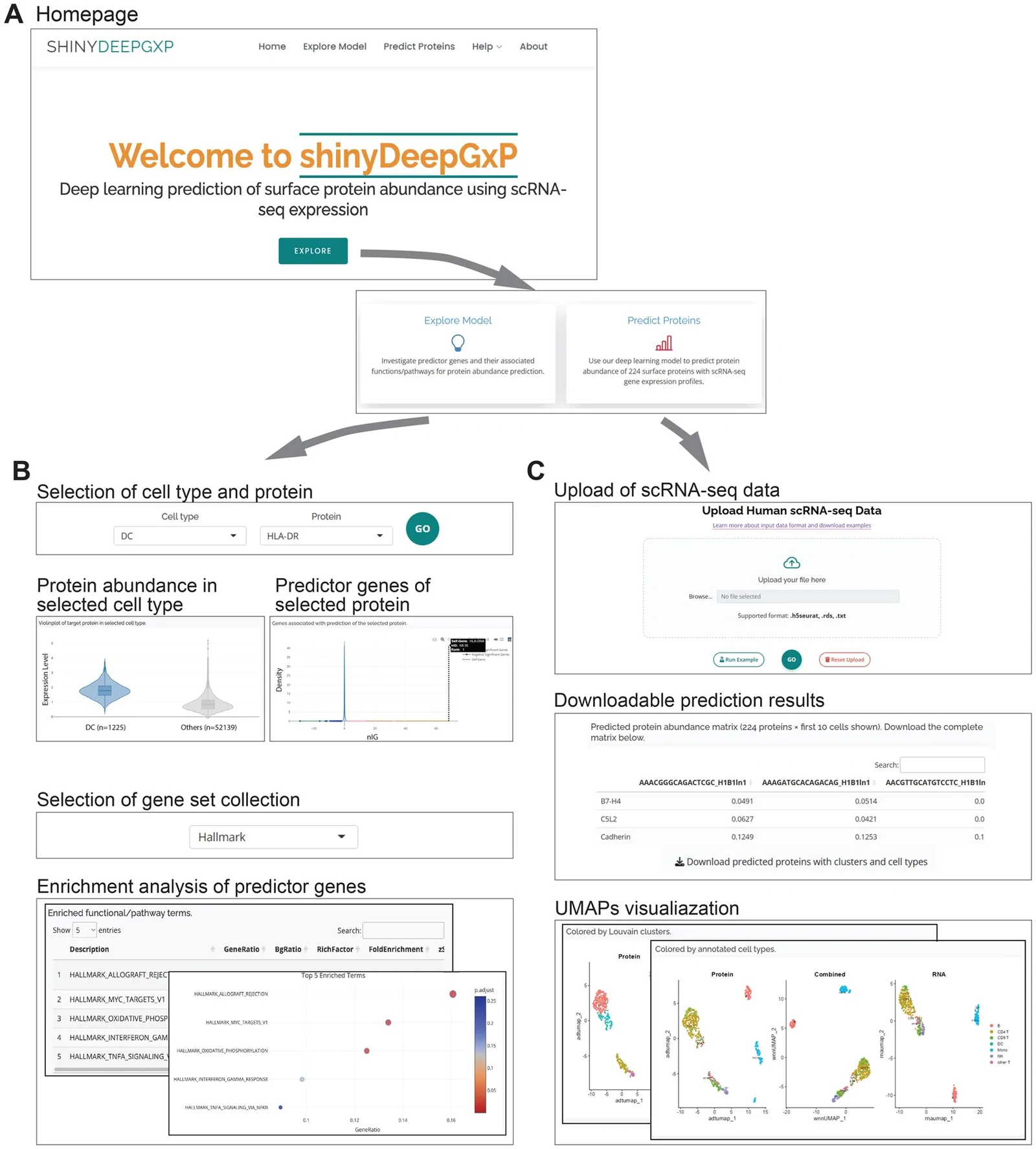
User interface of shinyDeepGxP. Screenshots of the main interface that illustrate the layout and functionality of the two modules. (A) Homepage of shinyDeepGxP summarizing the DeepGxP training dataset and providing access to the two interactive modules. (B) Interface of Module 1 (Explore Model). Users select a cell type and a target protein to explore model interpretation results. The output includes a violin plot comparing protein abundance in the selected cell type versus all other cells, a density plot showing the distribution of normalized integrated gradient (nIG) scores for predictor genes, and pathway enrichment analysis based on the selected gene set collection. (C) Interface of Module 2 (Predict Proteins). Users upload scRNA-seq data in.h5seurat format. The output displays predicted protein abundances alongside UMAP visualizations colored by Louvain clusters and by reference-based cell type annotations.

shinyDeepGxP provides an interactive web interface that enables users to predict surface protein abundance from scRNA-seq data using the DeepGxP model. The platform consists of two modules: Explore Model, which allows users to explore gene-level contributors underlying protein prediction, and Predict Proteins, which enables users to upload scRNA-seq data and generate predicted protein abundance profiles for downstream analysis. An overview of the shinyDeepGxP interface and workflow is shown in Fig. 2. The homepage serves as the entry point to the application and provides links to the two main modules (Fig. 2A). The Explore Model module enables exploration of protein-specific predictor genes and their associated enrichment results (Fig. 2B). The Predict Proteins module enables prediction of 224 surface proteins from uploaded scRNA-seq data and provides downstream visualization, clustering, cell type annotation, and downloadable outputs (Fig. 2C). Representative use cases for the two modules are described below.

### 3.1 Module 1: Model interpretation from DeepEnrich

Module 1 enables exploration of gene-level contributors underlying protein prediction using the DeepEnrich framework. To illustrate its functionality, we examined predictors associated with CD4 and CD20 protein abundances in CD4 T cells and B cells, respectively.

**Figure 3:**
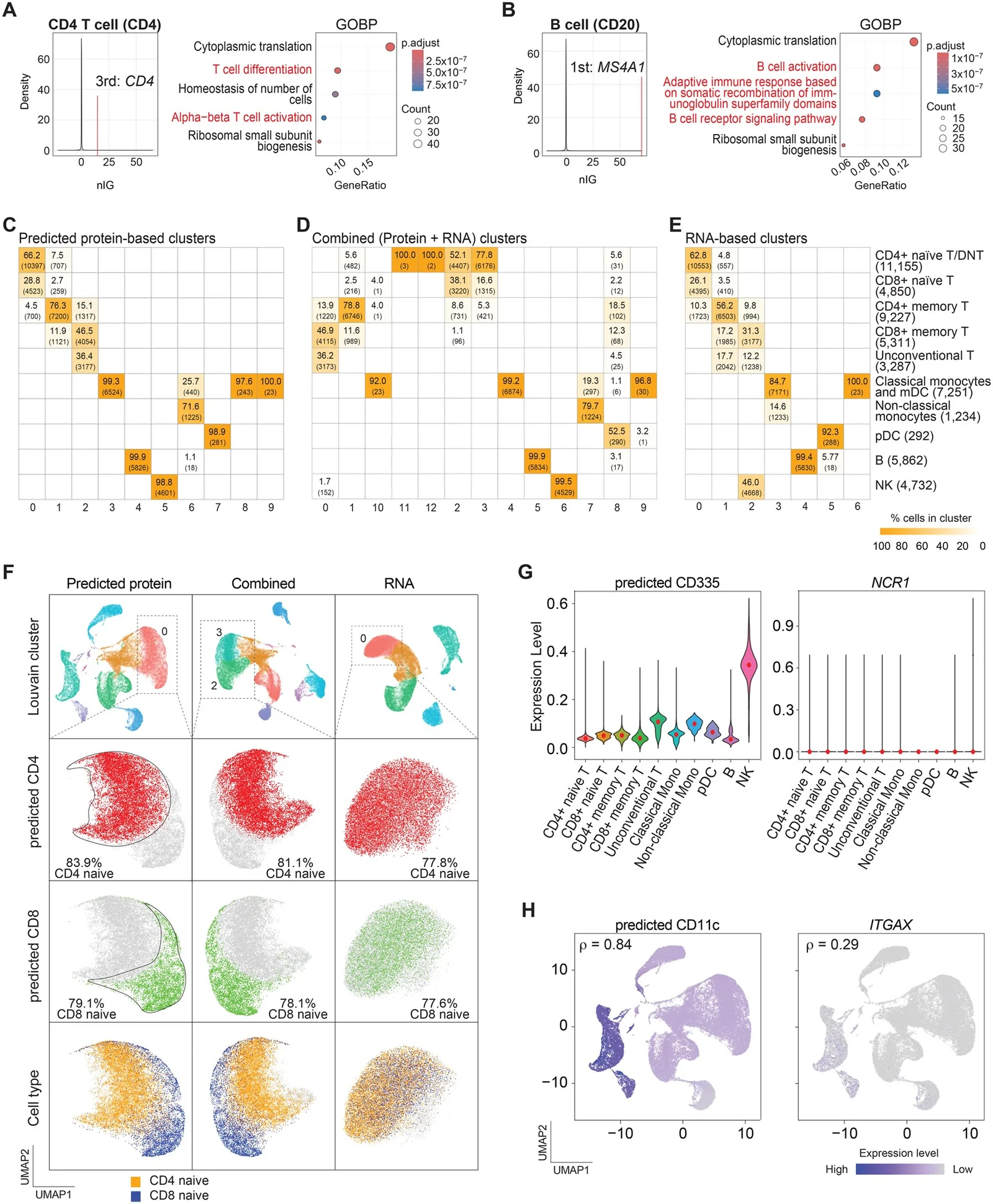
Interpretation of DeepGxP predictions and comparison of RNA and prediction protein features in single-cell analysis. Examples demonstrating model interpretation (Module 1) and downstream analysis based on predicted protein features (Module 2). (A) Interpretation of DeepGxP predictions for the CD4 protein in CD4+ T cells. Left: density distributions of normalized integrated gradient (nIG) scores showing gene-level contributions, with *CD4* highlighted. Right: GOBP enrichment analysis of the top predictor genes. (B) Interpretation of DeepGxP predictions for the CD20 protein in B cells. Left: density distributions of nIG scores *for MS4A1*, highlighted. Right: GOBP enrichment analysis of top predictor genes. (C–E) Heatmaps compare clustering results based on predicted protein expression (C), integrated RNA and protein features (D), and RNA expression alone (E), alongside reference cell type annotations from the original study. Louvain clusters are shown on the x-axis, and cell type labels on the y-axis. Numbers indicate the percentage of each cell type within a cluster relative to all cells in that cluster, with absolute cell counts shown below in parentheses. Only values ≥1% are displayed. Highlighted cells indicate strong correspondence between data-driven clusters and reference cell type labels. DNT: Double negative T cells. (F) UMAP projections based on predicted protein features (left, *n* = 224), integrated RNA and protein features (middle), and RNA features (right, *n* = 2000). From top to bottom, cells are colored by Louvain cluster assignments, predicted CD4 abundance, predicted CD8 abundance, and reference cell type annotation. In the first row, numbers indicate cluster identities, with labels shown only for clusters containing the majority of CD4 and CD8 naive T cells. Percentages in the second and third row indicate the percentage of CD4 naive and CD8 naive T cells among cells with high predicted CD4 or CD8 protein abundance, respectively. (G) Violin plots comparing the NK cell marker CD335 (predicted protein, left) and its encoding gene *NCR1* (RNA, right) across cell types. Red dots mark the median expression levels. (H) UMAP visualization of the monocyte marker CD11c, showing predicted protein abundance (left) and expression of its encoding gene *ITGAX* (RNA, right). ρ denotes the Pearson correlation between measured protein abundance and either predicted protein abundance or RNA expression.

In CD4 T cells, the density plot of normalized integrated gradient (nIG) scores identifies the *CD4* gene among the top-ranked predictors (Fig. 3A, left). Enrichment analysis of leading predictor genes highlights pathways related to T cell differentiation and alpha-beta T cell activation (Fig. 3A, right), consistent with known immunological functions. CD4 protein abundance is also elevated in CD4 T cells compared with other cell types (median = 5.68 versus 3.25; *P *< 2.2 × 10^−16^, Mann–Whitney *U* test).

A similar pattern is observed for the CD20 protein in B cells. *MS4A1*, the gene encoding CD20, is identified as the top-ranked predictor (Fig. 3B, left), with enriched pathways including B cell activation and B cell receptor signaling (Fig. 3B, right). CD20 protein abundance is likewise higher in B cells than in other cell populations (median = 1.31 versus 0.12; *P *< 2.2 × 10^−16^, Mann–Whitney *U* test).

These examples demonstrate how Module 1 links predicted protein abundance to gene-level contributions and functional enrichment, offering an interpretable view of the RNA-protein relationships learned by DeepGxP.

### 3.2 Module 2: Cell-type resolution from predicted protein features

To demonstrate the analytical capabilities of Module 2, we applied shinyDeepGxP to an independent H1N1 single-cell dataset (Kotliarov *et al*. 2020) and compared clustering results derived from predicted protein abundance, RNA expression, and combined modalities. The predictive performance of DeepGxP has been evaluated previously using multiple independent external datasets; therefore, the H1N1 dataset is presented here as a representative use case rather than an additional validation benchmark. Protein-based clustering, using 224 predicted proteins, produced more distinct cell type-specific clusters (*n* = 10) than RNA-based clustering using 2000 highly variable genes (*n* = 7) (Fig. 3C and E). For example, NK cells formed a distinct protein cluster (cluster 5, 98.8%), whereas they were partially mixed with other T cells in RNA cluster 2 (46.0%). Similarly, CD4^+^ memory T cells and classical monocytes were more clearly distinguished in protein clusters (cluster 1, 76.3% and cluster 6, 71.6%) compared with RNA-based clustering (clusters 1, 56.2% and cluster 3, 14.6%). Integrating RNA and predicted protein data further improved resolution, particularly for non-classical monocytes, increasing from 71.6% (protein) to 79.7% (combined; Fig. 3D).

UMAP visualization further highlighted differences between modalities. CD4^+^ and CD8^+^ naive T cells were more clearly separated in the protein space, whereas the RNA space showed dispersed overlap between these populations (Fig. 3F). Predicted protein abundance also followed expected marker patterns, with 79.1% of CD4-expressing cells corresponding to CD4 naïve T cells and 83.9% of CD8-expressing cells corresponding to CD8 naive T cells.

Module 2 also enables users to explore discrepancies between RNA and protein expression patterns. For instance, the NK cell marker CD335 showed strong cell-type specificity at the predicted protein level (median = 0.34), whereas its encoding gene, *NCR1*, showed minimal expression (median = 0; Fig. 3G). Similarly, predicted CD11c abundance showed clear monocyte-specific expression, whereas the gene *ITGAX* showed weak RNA expression (Fig. 3H). The Pearson correlation between measured CD11c protein and *ITGAX* RNA expression was modest (ρ = 0.29), whereas the correlation between measured and DeepGxP-predicted protein abundance was substantially higher (ρ = 0.84).

Together, these examples illustrate that predicted protein profiles generated by shinyDeepGxP complement transcriptomic data and support downstream analyses such as multimodal clustering, marker visualization, and cell-type annotation. By providing these capabilities within an interactive web platform, shinyDeepGxP facilitates exploration of RNA-protein relationships in single-cell datasets.

## 4 Discussion

Here we present shinyDeepGxP, an interactive web platform that uses the DeepGxP deep learning model to predict surface protein abundance from single-cell RNA expression. The platform enables users to analyse predicted protein profiles alongside transcriptomic features through clustering, visualization, and multimodal analyses, all within an accessible browser-based interface.

Case studies demonstrate that predicted protein abundances yield stronger cell-type signals than RNA expression alone, improving the resolution of immune populations, including CD4+ and CD8+ naive T cells, NK cells, and monocytes. In addition to prediction, shinyDeepGxP incorporates the DeepEnrich module, enabling users to examine gene-level contributions and the enriched biological pathways underlying model predictions. This interpretability component allows researchers to investigate the biological signals captured by the model rather than treating predictions as a black box.

Several limitations should be acknowledged. DeepGxP was trained on PBMC CITE-seq data, and therefore optimized for the immune cell populations represented in the training dataset. As a result, model performance is expected to be strongest for blood-derived immune cells (e.g. T cells, B cells, NK cells, and monocytes). When applied to datasets from other tissues or cell types, prediction accuracy may be reduced due to differences in underlying transcriptome-proteome relationships. Therefore, for out-of-domain datasets, predicted protein abundance should be interpreted cautiously. Future developments may include protein-based cell-type annotation and other protein-focused analyses, such as inferring ligand-receptor interactions, especially for surface proteins with weak RNA-protein correlations.

Overall, shinyDeepGxP offers an accessible framework for deriving protein-level insights from scRNA-seq datasets without needing extra experimental assays.

## Data Availability

The data and source code underlying this article are available in Figshare at https://doi.org/10.6084/m9.figshare.28875743. The shinyDeepGxP web server is available at https://shiny.crc.pitt.edu/deepgxp/.
